# Supplementary figures and images for: Population size, density, and ranging behaviour in a key leopard population in the Western Cape, South Africa
Source: PLoS One. 2022 May 27;17(5):e0254507. doi: 10.1371/journal.pone.0254507 (PMC9140237; doi:10.1371/journal.pone.0254507)

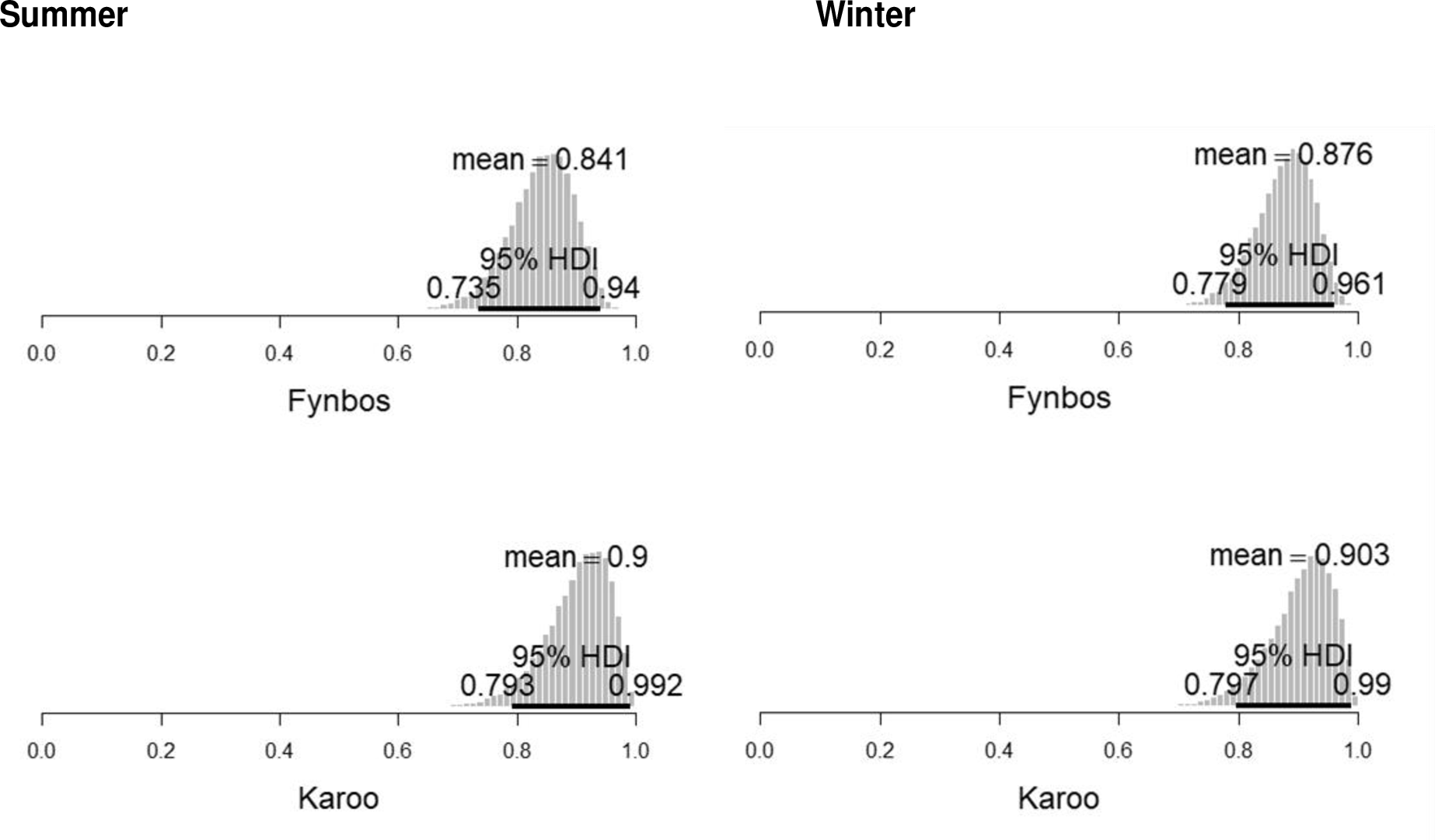

Supplement: S1 Fig — (TIF) [file pone.0254507.s003.tif]

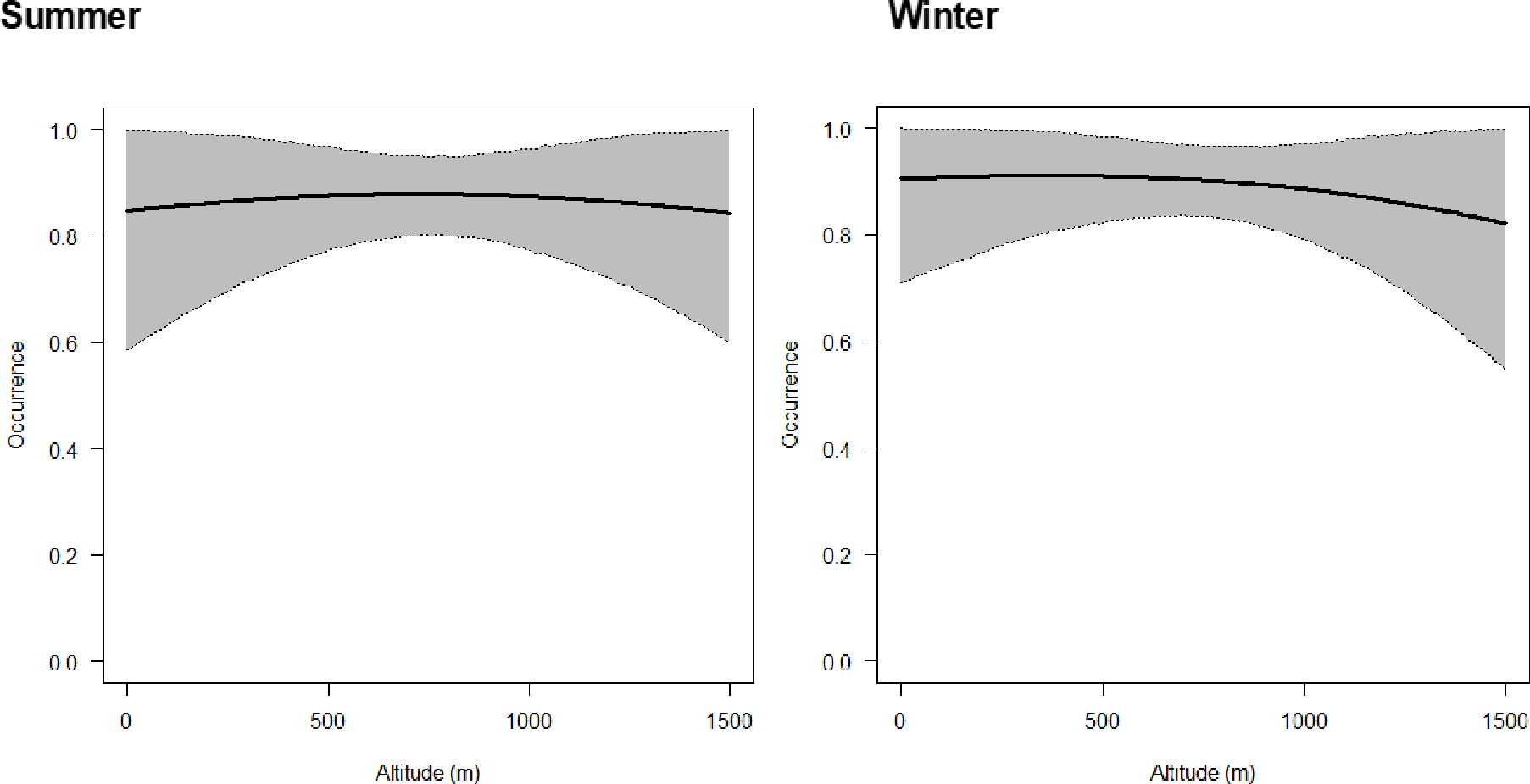

Supplement: S2 Fig — (TIF) [file pone.0254507.s004.tif]

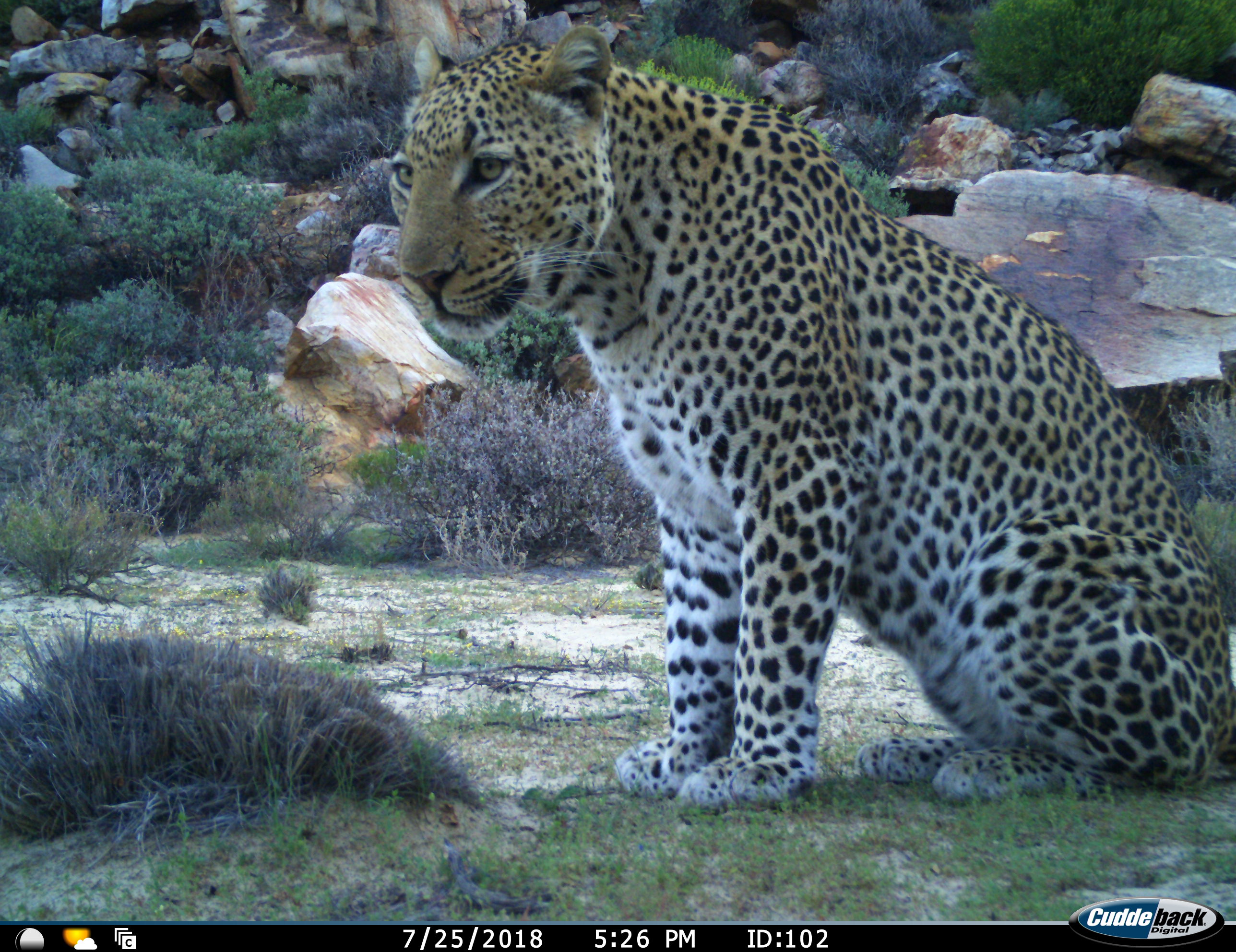

Supplement: S3 Fig — (JPG) [file pone.0254507.s005.jpg]
