# Supplementary material for: Population size, density, and ranging behaviour in a key leopard population in the Western Cape, South Africa
Source: PLoS One. 2022 May 27;17(5):e0254507. doi: 10.1371/journal.pone.0254507 (PMC9140237; doi:10.1371/journal.pone.0254507)
Supplement: Attachment — Submitted filename: REVIEWERS COMMENTS-KAHANA.docx [file pone.0254507.s006.docx]

**REVIEWER’S COMMENTS**

I reviewed the manuscript “POPULATION SIZE, DENSITY AND RANGING BEHAVIOUR IN A KEY LEOPARD POPULATION IN THE WESTERN CAPE, SOUTH AFRICA”. The manuscript certainly has merit for publication; it is well organized and written clearly, as it discusses leopard population and ranging behavior. In addition, the manuscript concludes that “ Effectively conserving leopards in a human-dominated landscape requires a good understanding of population estimates. The robust statistical approaches used in our study are important as they provide accurate population estimates that can be used to make informed management and conservation decisions at both local and regional scales. Leopard densities and ranging behaviour in the Western Cape indicate that large tracts of protected land and connectivity of the landscape are essential requirements for the long-term survival of leopard populations, as well as increased efforts in conflict mitigation, especially during winter months. Since the majority of the remaining suitable leopard habitat falls within non- protected areas, we suggest that conservation efforts should focus on privately-owned farmlands through the formation of conservancies”, but this conclusion is not supported by part of the findings.

Methods for data collections used are detailed enough and have addressed all the elements under study such as population size and density, site use, season livestock depredation and ranging behavior and the statistical tests used are sufficiently described.

Results are presented clearly, logically and sequentially in text, although I have some concerns about the camera trap location (GPS coordinates) are not shown anywhere in the tables presented. I suggested this information be presented as part of the results. This will allow an easy repetition of the study for someone wants to do it again.

Discussion has given a brief summary of important findings by discussing what the results obtained means in the context of hypothesis. The recommendations made within the discussion section starting at line 385 with the word however to line 388 and 430 to 439, fits well to be part of conclusion and recommendation section.

The conclusion is missing part of the findings discussed. It should summarize all the findings and provide recommendation for future work that is presented in the discussion section.
