# Supplementary material for: Population size, density, and ranging behaviour in a key leopard population in the Western Cape, South Africa
Source: PLoS One. 2022 May 27;17(5):e0254507. doi: 10.1371/journal.pone.0254507 (PMC9140237; doi:10.1371/journal.pone.0254507)
Supplement: Attachment — Submitted filename: Response to Reviewers.docx [file pone.0254507.s007.docx]

Response to Reviewers

Dear Academic Editor and Reviewers

Thank you for taking the time and making the effort to review our manuscript. We appreciate all the comments and feel that these will help the manuscript to be more suitable for publication in the PLOS ONE journal.

We have worked through all the comments and have responded to the best of our ability. All authors have read the comments, given their inputs and accepted the responses. Please see our responses to the comments below. We have also addressed these in the manuscript.

Academic Editor (AE)

**AE comment (1)**: Please ensure that your manuscript meets PLOS ONE's style requirements, including those for file naming.

**Response:** We have reviewed PLOS ONE’s style requirements, including those for file renaming and we believe we have corrected all inconsistencies.

**AE comment (2)**: Please include your tables as part of your main manuscript and remove the individual files. Please note that supplementary tables (should remain/ be uploaded) as separate "supporting information" files.

**Response:** We have included Table 1 within the main manuscript in our resubmission. As suggested by the reviewers, we have also included two supplementary tables (S1_ Table and S2_Table) as separate “supporting files” as per the Academic Editor’s request.

**AE comment (3)**: In your Methods section, please provide additional information regarding the field permits you obtained for the work. Please ensure you have included the full name of the authority that approved the field site access and, if no permits were required, a brief statement explaining why.

**Response:** A permit for camera trapping and scat collection was obtained from CapeNature, the governing body of nature conservation in the Western Cape Province of South Africa. The permit (No: 0056-AAA007-00217) was issued 25/4/2017 and expired 31/1/21.

**AE comment (4)**: We note that the grant information you provided in the ‘Funding Information’ and ‘Financial Disclosure’ sections do not match. When you resubmit, please ensure that you provide the correct grant numbers for the awards you received for your study in the ‘Funding Information’ section.

**Response:** We have checked and corrected this.

**AE comment (5)**: Thank you for stating the following in the Acknowledgments Section of your manuscript: “We are very grateful to our donors, Abax Foundation, Bushmans Kloof Wilderness Reserve, Lomas Wildlife Protection Trust, and the Rolf Stephan Nussbaum Foundation. Without their support this work would not have been possible. We thank Ford Wildlife Foundation for providing a vehicle for the research team. A special thanks also to Rika du Plessis from Cape Nature and all Cederberg landowners for their support and providing us access to their properties. We acknowledge the Cape Leopard Trust for providing the resources to enable this research and the Scientific Advisory Board for their input during the design phase of this study. We thank Gareth Mann for his guidance on selecting good camera trap sites for leopards and his assistance with the Camerabase software. We thank Ewan Brennan, Hannes de Kok, Ismail Wambi, Lizette Burger, James Wells, and Grant Baker for assisting with the camera trap survey and MJ Grobler, Louw Redelinghuys, and Ockie Müller for assisting with the data processing. Finally, we want to thank Professor Dan Parker and Dr Alison Leslie for reviewing this manuscript.We note that you have provided funding information that is not currently declared in your Funding Statement. However, funding information should not appear in the Acknowledgments section or other areas of your manuscript. We will only publish funding information present in the Funding Statement section of the online submission form. “

Please remove any funding-related text from the manuscript and let us know how you would like to update your Funding Statement. Currently, your Funding Statement reads as follows: “The work is supported by the Cape Leopard Trust whom received funding from Abax Foundation, Bushmans Kloof Wilderness Reserve, Lomas Wildlife Protection Trust, and the Rolf Stephan Nussbaum Foundation. Ford Wildlife Foundation has sponsored a vehicle for the research team.” Please include your amended statements within your cover letter; we will change the online submission form on your behalf.

**Response:** We have reworded the text in the manuscript in the Acknowledgments section to:

“We are very grateful to Abax Foundation, Bushmans Kloof Wilderness Reserve, Ford Wildlife Foundation, Lomas Wildlife Protection Trust, and the Rolf Stephan Nussbaum Foundation for their support. A special thanks also to Rika du Plessis from Cape Nature and all Cederberg landowners for their cooperation and providing us access to their properties. We acknowledge the Cape Leopard Trust for facilitating this research and the Scientific Advisory Board for their input during the design phase of this study. We thank Gareth Mann for his guidance on selecting good camera trap sites for leopards and his assistance with the Camerabase software. We thank Ewan Brennan, Hannes de Kok, Ismail Wambi, Lizette Burger, James Wells, and Grant Baker for assisting with the camera trap survey and MJ Grobler, Louw Redelinghuys, and Ockie Müller for assisting with the data processing. Finally, we want to thank Professor Dan Parker and Dr Alison Leslie for reviewing this manuscript.”

Additionally, we have reworded the Funding Statement to: “The work is supported by the Cape Leopard Trust in association with Abax Foundation, Bushmans Kloof Wilderness Reserve, Lomas Wildlife Protection Trust, Rolf Stephan Nussbaum Foundation and the Ford Wildlife Foundation.”

**AE comment (6)**: We note that Figure 1 in your submission contain [map/satellite] images which may be copyrighted. All PLOS content is published under the Creative Commons Attribution License (CC BY 4.0), which means that the manuscript, images, and Supporting Information files will be freely available online, and any third party is permitted to access, download, copy, distribute, and use these materials in any way, even commercially, with proper attribution. For these reasons, we cannot publish previously copyrighted maps or satellite images created using proprietary data, such as Google software (Google Maps, Street View, and Earth). For more information, see our copyright guidelines: <http://journals.plos.org/plosone/s/licenses-and-copyright>.

We require you to either (1) present written permission from the copyright holder to publish these figures specifically under the CC BY 4.0 license, or (2) remove the figures from your submission:

 a. You may seek permission from the original copyright holder of Figure 1 to publish the content specifically under the CC BY 4.0 license.

 We recommend that you contact the original copyright holder with the Content Permission Form (<http://journals.plos.org/plosone/s/file?id=7c09/content-permission-form.pdf>) and the following text:

“I request permission for the open-access journal PLOS ONE to publish XXX under the Creative Commons Attribution License (CCAL) CC BY 4.0 (<http://creativecommons.org/licenses/by/4.0/>). Please be aware that this license allows unrestricted use and distribution, even commercially, by third parties. Please reply and provide explicit written permission to publish XXX under a CC BY license and complete the attached form.”

Please upload the completed Content Permission Form or other proof of granted permissions as an ""Other"" file with your submission.

In the figure caption of the copyrighted figure, please include the following text: “Reprinted from [ref] under a CC BY license, with permission from [name of publisher], original copyright [original copyright year].

b. If you are unable to obtain permission from the original copyright holder to publish these figures under the CC BY 4.0 license or if the copyright holder’s requirements are incompatible with the CC BY 4.0 license, please either i) remove the figure or ii) supply a replacement figure that complies with the CC BY 4.0 license. Please check copyright information on all replacement figures and update the figure caption with source information. If applicable, please specify in the figure caption text when a figure is similar but not identical to the original image and is therefore for illustrative purposes only.

The following resources for replacing copyrighted map figures may be helpful:

 USGS National Map Viewer (public domain): <http://viewer.nationalmap.gov/viewer/>

The Gateway to Astronaut Photography of Earth (public domain): <http://eol.jsc.nasa.gov/sseop/clickmap/>

Maps at the CIA (public domain): <https://www.cia.gov/library/publications/the-world-factbook/index.html> and <https://www.cia.gov/library/publications/cia-maps-publications/index.html>

NASA Earth Observatory (public domain): <http://earthobservatory.nasa.gov/>

Landsat: <http://landsat.visibleearth.nasa.gov/>

USGS EROS (Earth Resources Observatory and Science (EROS) Center) (public domain): [http://eros.usgs.gov/#](http://eros.usgs.gov/)

Natural Earth (public domain): <http://www.naturalearthdata.com/>

Please review your reference list to ensure that it is complete and correct. If you have cited papers that have been retracted, please include the rationale for doing so in the manuscript text, or remove these references and replace them with relevant current references. Any changes to the reference list should be mentioned in the rebuttal letter that accompanies your revised manuscript. If you need to cite a retracted article, indicate the article’s retracted status in the References list and also include a citation and full reference for the retraction notice.

**Response:** We believe we have addressed the issue with Fig 1. We have removed the shapefile containing the copyrighted (and potentially sensitive) data and created a new Fig 1. We received the shapefiles including the Protected area polygons directly from CapeNature and we will attach a signed agreement between CapeNature and Cape Leopard Trust for the sharing of these files.

Reviewer #1

**Reviewer comment**: Fig.1, Please, include a reference map of Africa continent

**Response**: We have included a reference map of the African continent in the manuscript.

**Reviewer comment**: If you divided the area in 50 km2 grids, it seems that every cell will have (if square) a 7km side; I do not understand an interdistance of 2 km between cameras? It is non-sense to put more cameras in some cells, which will increase detectability in cells with more cameras. Please, justify. Include the grid in fig.1. Also provide a mean interdistance and a range between cameras, not only the minimum distance. Give the average range size for males and females.

**Response**: The camera trapping grid selected for our study was 50km^2^ (see Mann et al., 2014; Devens et al., 2018 for similar grid spacing also in the same province as our study). The selection of a camera trapping grid is typically based on the minimum home range of a female leopard (Balme et al., 2009). This prevents any gaps in the survey area and thus reduces the likelihood of an individual being ‘missed’ during the study. The minimum recorded home ranges of leopards in the Cederberg is 74km^2^ (Martins & Harris, 2013), although this was based on only four female leopards and so the ranges of some females could be smaller than that found previously. Therefore, we used a modest grid of 50km^2^. Camera trap locations were selected based on the presence of leopard signs (e.g. previous captures on camera traps, scat, tracks, scratch trees etc.). All grid cells had two camera trap stations, which was done to maximize the likelihood of capturing individuals or to put it another way, reducing the likelihood of missing an individual in ‘gaps’ in the survey (see Balme et al., 2009; Karanth et al., 2011; Strampelli et al., 2018). Five out of the 34 grid cells had 3 camera trap stations, this was done to replicate all sites from a previous survey for consistency. The mean inter camera distance was 2.78 km (range = 0.5–6). This information was included in the manuscript. Home range of leopards in the Cederberg ranges from 74–910km^2^, while mean female range is 117±29 km^2^ and male range is 313±108 km^2^ (Martins, 2010).

References

Mann G. Aspects of the ecology of leopards (Panthera pardus) in the Little Karoo, South Africa. PhD thesis, Rhodes University; 2014 [Cited 2021 January 4]. Available from: https://core.ac.uk/download/pdf/145047034.pdf

Devens CH, Hayward MW, Tshabalala T, Dickman A, McManus JS, Smuts B, et al. Estimating leopard density across the highly modified human-dominated landscape of the Western Cape, South Africa. Oryx; 2018; 1–12. https://doi.org/10.1017/S0030605318001473.

Martins Q, Harris S. Movement, activity and hunting behaviour of leopards in the Cederberg mountains, South Africa. Afr J Ecol. 2013;51: 571–579. https://doi.org/10.1111/aje.12068

Balme GA, Hunter LTB, Slotow R. Evaluating methods for counting cryptic carnivores. J Wildl Manag. 2009;73: 433–441. https://doi.org/10.2193/2007-368

Karanth KU, Nichols JD, Kumar NS. Estimating tiger abundance from camera trap data: field surveys and analytical issues. In: O’Connell AF, Nichols JD, Karanth KU, editors. Camera Traps in Animal Ecology: Methods and Analyses. Japan: Springer; 2011. pp. 97–117.

Strampelli P, Andresen L, Everatt KT, Somers MJ, Rowcliffe JM. Leopard Panthera pardus density in southern Mozambique: evidence from spatially explicit capture-recapture in Xonghile Game Reserve. Oryx. 2018;1–7.

Martins QE. The ecology of the leopard Panthera pardus in the Cederberg Mountains. PhD thesis, University of Bristol; 2010 [Cited 2020 December 23]. Available from: https://research-information.bris.ac.uk/ws/portalfiles/portal/34506234/535218.pdf

**Reviewer comment**: The 73 cameras were simultaneously placed from October 2017 to September 2018 (11 months)? Please, clarify. How many days were the cameras in the field?

**Response**: Yes, we had 73 camera trap stations (146 cameras) running simultaneously for the entire survey period from 1 October 2017 to 31 March 2018 (summer) and 1 April 2018 to 30 September 2018 (winter). The cameras were in the field for 365 days (1 October 2017 – 30 September 2018). Camera setup started on 8 June 2017, all cameras were up and running by 24 July 2017 and camera trap retrieval started on 4 October 2018 and the last camera was taken down on 3 November 2018.

**Reviewer comment**: SCR? Needs to be described at first citation.

**Response**: Agreed. We have described this at the first citation as follows: “Leopard population densities were estimated using Bayesian spatial capture-recapture (SCR) models. SCR models are relatively new methods which extend traditional capture-recapture models by incorporating auxiliary information about individual capture locations into the modelling framework to explicitly estimate the density of animal activity centres, with animal abundance as a derived parameter [37,38].

37. Borchers DL, Efford MG. Spatially Explicit Maximum Likelihood Methods for Capture-Recapture Studies. Biometrics. 2008; 64:377–85.

38. Royle JA, Gardner B. Hierarchial Spatial Capture-Recapture Models for Estimating Density from Trap Arrays. In: Camera Traps in Animal Ecology: Methods and Analyses. Springer; 2011. pp.163–90.

**Reviewer comment (Line 170)**: Why these periods were selected, justify or add some reference. Where the curves of Fig.2 asymptotic? Can you calculate the actual proportion of the population sampled if the curves reached an asymptote?

**Response**: We wanted to look at seasons as a covariant and looked at the 6 months with the lowest temperatures and highest rainfall and categorized that as winter and the months with the lowest rainfall and highest temperatures as summer, as described in the Methods sections.

We believe the reviewer is referring to Fig 3 and not Fig 2? If this is the case, the curves from Fig 3 are asymptotic in nature. In summer, 46 leopard individuals were captured while the estimated asymptote was 60 individuals. In winter, 49 leopard individuals were captured while the estimated asymptote was 81 individuals. In summer, 90% of the asymptote (55 individuals) were reached after 900 days. In winter the 90% asymptote (73 individuals) were reached after 2500 days.

**Reviewer comment**: Why a 5 days-period? A weekly interval would have more sense, justify.
**Response**: We were more cautious and used 5-day interval to ensure independence of observations. We have also used this interval in other studies so wanted to be consistent.

**Reviewer comment**: I guess that “depredation” is not a correct term applied to the ecology of organisms, maybe better using “predation”

**Response**: Response: The word “depredation” has been used extensively in literature to describe the predation of carnivores on livestock. We provide a few citations below which also use the term “depredation”.

References:

Kissui BM, Kiffner C, Konig HJ, Montgomery RA. Patterns of livestock depredation and cost-effectiveness of fortified livestock enclosures in northern Tanzania. Ecol Evol. 2019;9: 11420–11433.

LeFlore EG, Fuller TK, Tomeletso M, Stein AB. Livestock depredation by large carnivores in northern Botswana. Glob Ecol Conserv. 2019;18: e00592.

Rust NA, Marker LL. Cost of carnivore coexistence on communal and resettled land in Namibia. Environ Conserv. 2013;41: 45–53.

Hoffmann CF, Kissui BM, Montgomery RA. Spatial pattern analysis reveals randomness among carnivore depredation of livestock. Front Ecol Evol. 2019;7: 478.

**Reviewer comment**: I think that a more interesting question (rather than seasonal predation) is whether predation events increased/decreased along the study period (2009-2018). This was not analysed, and it should be. Maybe if decreasing, this could be a reason to lower persecution and should be discussed.

**Response**: We have included results on annual depredation events as well as a paragraph discussing these findings in the discussion section of the manuscript. In R we used a GLM to determine the trend annual depredation events. Although the model indicated that there was not a significant increase or decrease in depredation events over the years, the model had a low R-squared value of 0.25 meaning that the model did not fit the data well. Therefore, we did not include this model in the manuscript.

The results have been rewritten to:

“A total of 222 livestock depredation events were recorded during the study period. Mean annual livestock depredation events was 22.2±10.4 [SD], and although events were lowest in 2009 (n = 3) and highest in 2018 (n = 42), exploratory analysis did not indicate an increasing or decreasing trend in livestock depredation over time. The number of reported livestock depredation events by leopards differed significantly between seasons (Z = 3.3, p < 0.01). Mean monthly depredation events reported during summer were 1.4 (max = 4) and 2.4 (max = 10) during winter, while mean number of livestock killed seasonally was 7.7 (range = 2–12) during summer and 14.9 (range = 1–38) during winter.”

The discussion section reads as follows:

“We found that reported livestock depredation events did not show a particular trend over time. This could be due to inconsistent reporting as data collected is reliant upon landowners reporting their losses to the relevant authorities and if landowners are not incentivised, reporting might not be their priority. This observed variation in depredation events over the years could also be influenced by leopard occupancy across the landscape over time [66]. Nevertheless, fewer leopards are killed annually compared to before conservation efforts commenced in 2008 [11] and so engaging with landowners to improve tolerance towards leopards and reducing livestock depredations through mitigation measures may be key in promoting human-leopard coexistence in the region.”

References

11. Martins QE. The ecology of the leopard Panthera pardus in the Cederberg Mountains. PhD thesis, University of Bristol; 2010 [Cited 2020 December 23]. Available from: https://research-information.bris.ac.uk/ws/portalfiles/portal/34506234/535218.pdf

66. Verschueren S, Briers-Louw WD, Torres-Uribe C, Siyaya A, Marker L. Assessing human conflicts with carnivores in Namibia’s eastern communal conservancies. Hum Dimens Wildl. 2020;1–16. https://doi.org/10.1080/10871209.2020.1758253

**Reviewer comment**: Despite the use of Poisson distribution can be right, maybe the low number of predation events (a lot of zeroes) can produce underdispersion issues. The use of the negative binomial sometimes is a better solution, but this might be checked.

**Response**: When running tests to determine whether the model had dispersion issues, we found that the dispersion parameter ratio was 0.926. Additionally, we investigated the median deviance residual which was close to zero, meaning that our model is not biased in one direction (i.e. no under- or over-dispersion).

**Reviewer comment**: I think that is necessary to have all the records obtained available to the readers (photos/contacts per species). Please, include it in supplementary material.

**Response**: We have included the number of independent captures per species for both seasons in a supplementary table (S2_Table).

**Reviewer comment**: Numbers < 10 in words.

**Response**: Agreed. We have changed these numbers to words.

**Reviewer comment**: Mean trap rates need some dispersal statistics

**Response**: This was an error on our part. This was not mean trap rate, rather overall trap rate. This is the total independent captures of leopards divided by the total number of trapping days, multiplied by 100. Therefore, it is a single value and we cannot show dispersion statistics for this. We have provided trapping rate for both seasons as well.

Reviewer #2

**Reviewer comment**: Since the majority of the remaining suitable leopard habitat falls within non-protected areas, we suggest that conservation efforts should focus on privately-owned farmlands through the formation of conservancies”, but this conclusion is not supported by part of the findings.

**Response**: Agreed. We have re-phrased this section as follows:

“Protected areas play an important role in the conservation of leopard populations, although the majority of the remaining suitable leopard habitat in South Africa falls within non-protected areas [8]. Therefore, it is crucial that buffer zones around protected areas and dispersal corridors among areas with suitable habitat are maintained, which will also require human-wildlife conflict mitigation, to ensure the long-term conservation of the South African leopard population [8].”

**Reviewer comment**: Results are presented clearly, logically and sequentially in text, although I have some concerns about the camera trap location (GPS coordinates) are not shown anywhere in the tables presented. I suggested this information be presented as part of the results. This will allow an easy repetition of the study for someone wants to do it again.

**Response**: We agree that the camera trap locations could be included in the manuscript submission. So we have decided to include the camera trap locations as a supplementary Table (S1_Table) in our resubmission.

**Reviewer comment**: Discussion has given a brief summary of important findings by discussing what the results obtained means in the context of hypothesis. The recommendations made within the discussion section starting at line 385 with the word however to line 388 and 430 to 439, fits well to be part of conclusion and recommendation section.

**Response**: Agreed. We have moved these recommendations to the conclusions section. See response below.

**Reviewer comment**: The conclusion is missing part of the findings discussed. It should summarize all the findings and provide recommendation for future work that is presented in the discussion section.

**Response**: We have re-phrased the conclusion section to summarize all the findings and to provide recommendation for future work. It currently reads as follows:

“Effectively conserving leopards in a human-dominated landscape requires a good understanding of population estimates. The robust statistical approaches used in our study are important as they provide accurate population estimates that can be used to make informed management and conservation decisions at both local and regional scales. Low leopard density and large ranging behaviour indicate that large tracts of protected land and connectivity of the landscape, as well as increased conflict mitigation efforts, especially during winter months when male leopard activity centres shift towards surrounding farmland and livestock depredation events increase, are essential requirements for the survival of this leopard population [54]. Our study found that leopards were homogenously distributed across the Cederberg study site and were unaffected by altitude, habitat or season. However, we recommend future studies to quantify the density gradient of leopards between protected and non-protected areas to evaluate the anthropogenic impact on leopard populations. Protected areas play an important role in the conservation of leopard populations, although the majority of the remaining suitable leopard habitat in South Africa falls within non-protected areas [8]. Therefore, it is crucial that buffer zones around protected areas and dispersal corridors among areas with suitable habitat are maintained, which will also require human-wildlife conflict mitigation, to ensure the long-term conservation of the South African leopard population [8]. We encourage future research to investigate the potential interconnectivity between the South African leopard populations and whether the corridor networks require protection and management.”

Yours sincerely,

Lana Müller
